# Correlates of Near-Infrared Spectroscopy Brain–Computer Interface Accuracy in a Multi-Class Personalization Framework

**DOI:** 10.3389/fnhum.2015.00536

**Published:** 2015-09-30

**Authors:** Sabine Weyand, Tom Chau

**Affiliations:** ^1^PRISM Laboratory, Bloorview Research Institute, Holland Bloorview Kids Rehabilitation Hospital, Toronto, ON, Canada; ^2^PRISM Laboratory, Institute of Biomaterials and Biomedical Engineering, University of Toronto, Toronto, ON, Canada

**Keywords:** near-infrared spectroscopy, brain–computer interface, personalized tasks, multi-class, correlation analysis

## Abstract

Brain–computer interfaces (BCIs) provide individuals with a means of interacting with a computer using only neural activity. To date, the majority of near-infrared spectroscopy (NIRS) BCIs have used prescribed tasks to achieve binary control. The goals of this study were to evaluate the possibility of using a personalized approach to establish control of a two-, three-, four-, and five-class NIRS–BCI, and to explore how various user characteristics correlate to accuracy. Ten able-bodied participants were recruited for five data collection sessions. Participants performed six mental tasks and a personalized approach was used to select each individual’s best discriminating subset of tasks. The average offline cross-validation accuracies achieved were 78, 61, 47, and 37% for the two-, three-, four-, and five-class problems, respectively. Most notably, all participants exceeded an accuracy of 70% for the two-class problem, and two participants exceeded an accuracy of 70% for the three-class problem. Additionally, accuracy was found to be strongly positively correlated (Pearson’s) with perceived ease of session (ρ = 0.653), ease of concentration (ρ = 0.634), and enjoyment (ρ = 0.550), but strongly negatively correlated with verbal IQ (ρ = −0.749).

## Introduction

### Near-infrared spectroscopy brain–computer interface

Brain–computer interfaces (BCIs) can be used as an access pathway for individuals with severe motor impairments as they require only brain activations and no muscular control (Allison et al., 2007). Near-infrared spectroscopy (NIRS) has recently gained attention as a BCI access modality due to its non-invasive extraction methods, gel-less donning, and robustness to electrical noise (Ferrari et al., 2004; Scholkmann et al., 2014). In general, NIRS can be used to detect changes in the amount of oxygen in neuronal blood, which reflect changes in brain activation (Boas et al., 2014; Scholkmann et al., 2014; Strait and Scheutz, 2014). A computer can be trained to discriminate between mental tasks based on changes of the hemodynamic response resulting from the task being performed.

Currently, most hemodynamic BCIs use two prescribed tasks to achieve binary control of a computer. When using binary BCIs for communication, these tasks can be mapped to a “scroll and select” or “yes and no” output (Naito et al., 2007). A handful of studies have been conducted on NIRS–BCIs over the prefrontal cortex (PFC), achieving average accuracies ranging from around 60 to 80% (Power et al., 2010, 2011; Naseer and Hong, 2013; Schudlo et al., 2013).

Multi-class BCIs (beyond binary) have the potential to provide users with more outputs, thereby increasing the rate of communication (Shin et al., 2013). However, as the number of classes increases, so will the difficulty in discriminating between classes. To date, limited research on multi-class NIRS–BCIs has been conducted. To the best of our knowledge, three studies have explicitly explored multi-class NIRS–BCIs over the PFC that could potentially be used for active computer control, namely Hirshfield et al. (2009), Power et al. (2012a), and Herff et al. (2013a). Herff et al. (2013a) classified mental workload states, using the n-back task with an average three-class and four-class accuracy of 50.3 and 44.5%, respectively. Hirshfield et al. (2009) discriminated between different levels of mental workloads, achieving an average three-class accuracy of 54%. Finally, Power et al. (2012a) were able to distinguish between mental math (MM), mental singing, and rest with an average accuracy of 56.2%. A second study by Herff et al. also explored differentiating mental arithmetic, word generation (WG), and mental rotation. Although the accuracies for a three-class problem were not explicitly stated, the authors indicated that the three-class accuracies were greater than chance (Herff et al., 2013b). Overall, these studies demonstrate proof-of-concept for a multi-class NIRS–BCI over the PFC, but are not at the level that is required for effective BCI use. It appears that none of the participants in these studies exceeded the 70% threshold, often cited as required for BCI control (Kübler et al., 2001).

### Personalized tasks

One potential method for improving the classification accuracies achievable in an NIRS–BCI is the use of researcher-selected personalized mental tasks, an approach whereby a user tries a variety of tasks and subsequently a subset of tasks that are most suitable for that user are selected by the researcher. Task selection is usually based on the discriminating power of the tasks. To date, personalized mental tasks have been explored in a two-class offline NIRS–BCI study (Weyand et al., 2015b), as well as in a two-class magnetic resonance imaging (MRI) BCI study (Sorger et al., 2009) and in two-class (Palaniappan, 2006), three-class (Chai et al., 2012), and four-class (Dobrea and Dobrea, 2009) electroencephalography (EEG) BCI studies. Overall, these studies conclude that there is significant inter-subject variability in brain activation elicited by the same mental tasks and cognitive processes, and as a result, the tasks that are most effective for controlling a BCI vary among users. Therefore, there is potential to improve classification accuracies by choosing the most discriminating tasks for each user (Palaniappan, 2006; Dobrea and Dobrea, 2009; Sorger et al., 2009; Chai et al., 2012; Weyand et al., 2015b). To the best of our knowledge, to date, personalized tasks have not been explored in an NIRS–BCI beyond the binary paradigm.

### Correlation between BCI accuracy and user characteristics

Another sparsely explored area in the literature is the prediction of BCI accuracy based on user characteristics, such as demographic traits, IQ, and state of mind. Determining the correlation between user characteristics and performance may help to reduce some of the large inter-subject variability in classification accuracies, steer future BCI development, and provide additional measures for selecting user-specific tasks.

To date, limited accuracy-user correlation research has been conducted in the field of NIRS–BCIs. However, several studies have examined the inter- and intra-subject correlations between accuracy and characteristics of able-bodied participants using various EEG-based BCIs. Studies have reported increased accuracy to be correlated with the following: increasing self-reported task enjoyment (Pearson’s ρ = 0.3, *p* < 0.1) (Friedrich et al., 2013), increasing challenge (Spearman’s ρ = 0.8, *p* < 0.01) (Kleih et al., 2011), decreasing sleep (Mann–Whitney test*, p* < 0.05) (Guger et al., 2009), increasing mood (multiple regression *b* = 0.498, *p* < 0.05) (Nijboer et al., 2008), increasing mastery confidence (multiple regression *b* = 0.578, *p* < 0.05) (Nijboer et al., 2008), and increasing attention (Spearman’s ρ = 0.5, *p* = 0.02) (Hammer et al., 2012). Conflicting trends have been reported on the association of accuracy with fear of incompetence, i.e., anxiety of failing the task. Studies have noted increased accuracy with increasing (Spearman’s ρ = *0*.37, *p* < 0.05) (Kleih et al., 2011), and decreasing (multiple regression *b* = −0.616, *p* < 0.05) (Nijboer et al., 2008) fear of incompetence, both when visual feedback was provided. However, in the presence of auditory feedback accuracy and fear increased together (multiple regression *b* = 0.47, *p* < 0.05) (Nijboer et al., 2008).

Limited research has also been conducted on the correlation of accuracy with user demographics. Randolph et al. (2006) documented a positive relationship between age and control signal strength (*multiple linear regression*, *p* = 0.013). Meanwhile, Allison et al. (2010) observed that older subjects and male subjects tended to perform worse; however, it is noted that these trends were not significant.

In contrast to the above, several researchers found no correlations between accuracy and user characteristics; for example, in Guger et al. (2009), gender, education, work duration, and cigarette and coffee consumption were not statistically related to accuracy, and in Hammer et al. (2012), intelligence, mood, motivation, or learning abilities were not correlated with accuracy.

In addition to the studies on able-bodied participants, Nijboer et al. performed an intra-subject correlation analysis on six individuals with amyotrophic lateral sclerosis to explore the effect of quality of life, depression, mood, mastery confidence, incompetence fear, interest, and challenge on performance over time (across sessions). They found that BCI performance was positively related to mastery (Spearman’s ρ = + 0.805) in one participant, positively related to challenge (Spearman’s ρ = + 0.733) in another, and negatively related to incompetence fear (Spearman’s ρ = −0.824) in a third. No other correlations were found in the remaining three participants (Nijboer et al., 2010).

Although, to the best of our knowledge, no studies have explored correlations of NIRS–BCI performance with respect to user characteristics, the variety of correlations reported in EEG-BCI literature, along with the known dependencies of neural oxygenation levels on gender (Okada et al., 1993), handedness (Okada et al., 1993), age (Kwee and Nakada, 2003; Schroeter et al., 2003), and IQ (Graham et al., 2010), suggest that such an investigation is warranted.

### Objectives

The first objective of this study was to use a personalized mental task approach to determine the accuracies achievable for a two-, three-, four-, and five-class NIRS–BCI. The second objective was to ascertain the strength of the correlations between the accuracy achieved over five sessions by each participant and his or her personal characteristics, specifically, gender, handedness, age, verbal IQ score, average self-reported ease of session, average self-reported session enjoyment, average self-reported user tiredness, and average self-reported ease of concentration.

## Materials and Methods

It is noted that the data collected during this study were also analyzed to compare two-class prescribed and personalized NIRS–BCI frameworks offline. For more information on this work, please refer to Weyand et al. (2015b).

### Participants

Ten able-bodied participants (four males, six females) were recruited from the staff and students at Holland Bloorview Kids Rehabilitation Hospital (Toronto, Canada). Signed consent was obtained from all participants and the study was approved by the ethics departments at Holland Bloorview Kids Rehabilitation Hospital and the University of Toronto. All participants were self-selected and naïve to NIRS–BCIs.

### Criteria

Participants had normal or corrected-to-normal vision, were not receiving psychoactive medication such as anti-depressants or analgesics, and did not have any health conditions that may affect the measurement of or one’s ability to perform mental tasks, including, but not limited to, degenerative disorders, cardiovascular disorders, metabolic disorders, trauma-induced brain injury, respiratory conditions, drug and alcohol-related conditions, and psychiatric disorders. Lastly, participants had to communicate in English, refrain from smoking, and avoid drinking alcohol or caffeinated beverages 3 h prior to data collection.

### Instrumentation

**Figure 1:**
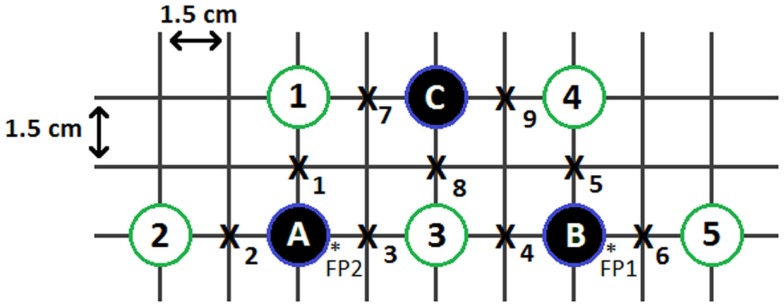
**Experimental source and detector configuration**. The solid circles represent detectors; the open circles represent light source pairs; the x’s represent points of interrogation (channels); and the starred areas represent the approximate FP1 and FP2 positions of the international 10–20 EEG system.

The Imagent Functional Brain Imaging System from ISS Inc., Champaign, IL (ISS Inc, 2012) was used to collect the NIRS data at a sampling rate of 31.25 Hz. Three photomultiplier tube detectors and five laser diodes (emitting 690 and 830 nm light) were arranged in a trapezoid, as shown in Figure 1. The trapezoid configuration allowed for discrete signal extraction at nine points of interrogation, located between each detector and diode that is separated by a distance of 3 cm (Naito et al., 2007). A headband made of rubber polymer (3M 9900 series) was used to position the detectors and light sources. All detectors and diodes were held in place by opaque fabric pockets that provided shielding from ambient light, and minimized detector and diode motion, while maximizing contact with the head. The headband was positioned above the eyebrows and centered with respect to the nose. Additionally, an accelerometer attached to the headband was used to collect information on head movement.

### Experimental protocol

#### Study Structure and User Interface

Participants took part in five data collection sessions on five separate days. Each session consisted of three data collection blocks. During each block, a baseline of 30 s was collected followed by 24 task intervals. The task intervals consisted of a task being performed for 20 s followed by a 17 s rest period. All six tasks were performed four times in a random order. Participants were asked to remain still during the task intervals.

Each task was performed 60 times by each participant (4 repetitions/block × 3 blocks/session × 5 sessions = 60).

**Figure 2:**
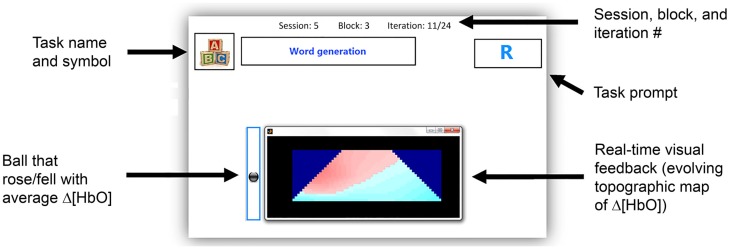
**User interface for all sessions**. The task name and symbol show which of the six tasks the user should perform, i.e., mental math, word generation, happy thoughts, relaxing with focus, relaxing with slow counting, or unconstrained rest.

Participants were provided with two forms of feedback as follows: (1) a real-time trapezoidal topographic image that corresponded to the hemodynamic changes over the entire interrogation area and (2) a ball that rose and fell depending on the average activation over the trapezoid. The goal of the neurofeedback was to provide participants with real-time information about changes in their hemodynamic activity when performing each of the tasks. Participants were informed that they should not stop performing the tasks; however, they could slightly modify the tasks, i.e., perform the tasks faster or slower, in order to try and achieve a more consistent change in the feedback. In a study by Schudlo and Chau, it was found that 8 out of 10 participants adjusted their mental strategies when using feedback (Schudlo and Chau, 2014).The feedback was updated every 125 ms, and was calculated using cubic interpolation of the oxygenated hemoglobin (HbO) values at equally spaced intervals between the points of interrogation. The topographic image was 21 pixels in height with parallel sides of 21 and 61 pixels in length, as in Schudlo and Chau (2014) and Weyand et al. (2015a). The red color on the feedback represented an increase in hemodynamic activity, while the blue color represented a decrease in hemodynamic activity. The user interface, including the two types of feedback, is shown in Figure 2.

#### Mental Tasks

In this study, we explored six mental tasks, selected on the basis of past literature indicating their suitability for NIRS–BCI control. Each of the six mental tasks is described in Table 1.

**Table 1:** **Mental tasks and descriptions**.

Mental task	Description
Mental math (MM)	Users continuously subtract a randomly generated two-digit number from a randomly generated three-digit number. For example, given the equation “593-11,” users would think “593-11 = 582; 582-11 = 571; 571-11 = 560…” (Naito et al., 2007; Ogata et al., 2007; Utsugi et al., 2007; Bauernfeind et al., 2008; Power et al., 2010, 2011, 2012b; Ang et al., 2012; Herff et al., 2013b; Schudlo et al., 2013; Schudlo and Chau, 2014)
Word generation (WG)	Users think of as many words that start with a randomly generated letter. For example, given the letter “D.” users may think of “dog, data, dashboard, donut…” (Ogata et al., 2007; Utsugi et al., 2007)
Happy thoughts (HT)	Users think of a past event in their life that made them happy (Tai and Chau, 2009)
Relaxing with focus (RF)	Users concentrate on the trapezoid activation feedback. Focusing on either the increasing or decreasing portions (Izzetoglu et al., 2011)
Relaxing with slow counting (RS)	Users count slowly, starting from any number that they wish (Naseer and Hong, 2013)
Unconstrained rest (RR)	Users are allowed to let their mind wander and may think of anything other than the five mental tasks (Naito et al., 2007; Power et al., 2011, 2012b; Ang et al., 2012; Herff et al., 2013b; Schudlo et al., 2013; Schudlo and Chau, 2014)

### Additional data collection

The Ammons Quick Test was used to asses verbal IQ. The Ammons Quick Test is a 5–15 min standardized verbal IQ test designed by Robert and Carol Ammons in 1962 and was administered after the last data collection session. The test consists of 50 questions in which users are asked to attribute a given word with one of four given pictures. The Ammons Quick Test has been shown to provide a good approximation of the full-scale IQ as measured by the Wechsler intelligent scale for adults (WAIS) with Pearson’s product moments of 0.85 (Zagar et al., 2013) and 0.89 (Husband and DeCato, 1982). The Ammons Quick Test has been used in several psychiatric studies (Advokat et al., 2005; Marjoram et al., 2005). It should be noted that one of the 10 participants (P4) chose not to complete the Ammons Quick Test.

A background questionnaire was administered prior to data collection to collect demographic data on each participant, including the participant’s age range, gender, and handedness.

A post-session questionnaire was completed at the end of each session. Participants evaluated the following subjective statements on a 7-point Likert-type scale ranging from “Strongly Agree” to “Strongly Disagree”: (1) I was tired before the session began, (2) I found it easy to concentrate during the session, (3) the session was fun, (4) it was easy to perform the tasks and session, and 5) the headgear was comfortable. For correlation analysis, the answers from all five post-session questionnaires were averaged for each participant.

### Data analysis

As a result of observed participant head motion or loss of contact between the head and the detectors, up to 20 data points (a maximum of four per class) were discarded from participants 3 and 9.

#### Filtering

The NIRS data were filtered in order to minimize noise due to the Mayer wave at a frequency of 0.1 Hz, the respiration cycle at a frequency of 0.2–0.4 Hz, and the cardiac cycle at a frequency of 0.5–2.0 Hz. A low-pass third-order Chebyshev infinite impulse response (IIR) cascade filter was used with a pass-band from 0 to 0.1 Hz, a transition band from 0.1 to 0.5 Hz, a stop-band from 0.5 Hz onward, and a pass band ripple of 0.1 dB.

#### Calculating Hemoglobin Concentrations

The modified Beer Lambert’s law (Delpy et al., 1988) was used to calculate changes in the concentrations of deoxygenated hemoglobin (Δ[Hb]), oxygenated hemoglobin (Δ[HbO]), and total hemoglobin (Δ[tHb]), as in (Power et al., 2012b; Schudlo et al., 2013; Schudlo and Chau, 2014; Weyand et al., 2015a,c).

#### Feature Extraction

Features extracted from the NIRS signal consisted of the temporal and spatial changes in the concentrations of the three chromophores (Hb, HbO, and tHb) over the four time windows (0–5s, 0–10s, 0–15s, and 0–20s). Specifically, the temporal features consisted of the linear regression slope over the normalized time windows for each of the 9 points of interrogation (4 time windows × 3 chromophores × 9 points of interrogation = 108 features), and the spatial features consisted of the linear regression slope over the zero- to fourth-order discrete orthogonal Chebyshev image moments over the four time windows (4 time windows × 3 chromophores × 15 image moments = 180 features). For more information on the extracted features, please refer to Schudlo et al. (2013) and Weyand et al. (2015b). Three distinct feature sets were considered during this study: temporal features only (108 features), spatial features only (180 features), and temporal combined with spatial (temporal–spatial) features (288 features).

#### Feature Selection

For each of the three distinct feature sets, a subset of the features was selected from the training data to reduce the dimensionality of the problem and reduce redundancy. The fast correlation-based filter (FCBF) was implemented (Yu and Liu, 2003). FCBF is useful for feature sets with high dimensionality and has been used previously in EEG-BCI studies (Chanel et al., 2007; Koelstra et al., 2010) and in the detection of the hemodynamic response by MRI (Tripoliti et al., 2007). For more information on the FCBF, please refer to Yu and Liu (2003). The FCBF typically reduced the high-dimensional feature sets to subsets consisting of 3–5 features.

## Data Analysis

### Offline classification

Offline classification accuracies were calculated using 10 iterations of 10-fold cross-validation (Refaeilzadeh et al., 2009). For each iteration of 10-fold cross-validation, the data were randomly separated into 10 equal sized portions (folds). Ten classification accuracies were calculated by iteratively using each fold as testing data and the remaining folds as training data. Only training data were used for feature selection and classifier training, and only the testing data were used to estimate the classification accuracies. Finally, all classification accuracies were averaged to estimate the overall accuracy.

### Multi-class classification algorithm

Classification was performed for all possible *n*-class combinations of the six mental tasks (where *n* = 2, 3, 4, or 5 classes). Specifically, the 6 choose *n* (*_6_C_n_*) task combinations for the 2-, 3-, 4-, and 5-class problems resulted in a total of 15, 20, 15, and 6 unique task combinations being explored for each classification problem, respectively.

Multi-class classification was conducted in a one-vs.-one (OVO) manner by simplifying each *n*-class problem into *m* binary problems (where *m* = *_n_C_2_*) and voting on the majority class (Dietterich and Bakiri, 1995; Rocha and Goldenstein, 2014). The number of binary problems (*m*) given *n* = 2, 3, 4, and 5 classes, were 1, 3, 6, and 10, respectively.

The class of each of the *m* binary classifiers was determined by the majority vote of three ensemble classifiers, one for each feature set (temporal features only, spatial features only, and temporal and spatial features). In particular, a bagging ensemble classifier with 10 members of linear discriminant analysis classifiers was used for each feature set.

**Figure 3:**
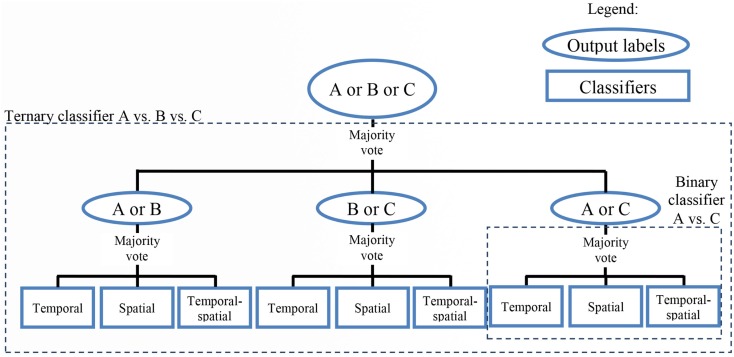
**Classification scheme for a sample three-class problem**. The output of each of the binary (two-class) classifiers was determined by a majority vote of the three ensemble classifiers (temporal, spatial, and temporal–spatial). Subsequently, the output of the ternary (three-class) classifier was derived from the majority vote of the three binary classifiers.

Figure 3 shows an example of the classification algorithm for one set of three tasks (Task A vs. Task B vs. Task C). The label for the testing data was predicted by a majority vote of the three binary classifiers (A vs. B, A vs. C, and B vs. C), whose individual outputs were derived from a majority vote of three ensemble classifiers (temporal, spatial, and temporal-spatial).

### Correlations between accuracy and user characteristics

The normality of the data was confirmed with the Shapiro–Wilk normality test. Pearson’s correlations with *α* = 0.1 were computed between accuracy and user characteristics following normal distributions, including IQ and state of mind data. Spearman’s Rho correlations with *α* = 0.1 were computed between accuracy and the demographic data. For brevity, only the correlations with respect to the best two-class accuracies were reported. The alpha value for the correlation analyses was set to 0.1 to minimize type II errors – i.e., missing a correlation that exists. Although this increases the probability of type I errors – i.e., finding a correlation when there is not one – at this stage, we believe it is more important to find potential correlations (Banerjee et al., 2009). Moreover, a similar value has been used in a previous EEG-BCI correlation analysis (Friedrich et al., 2013).

## Results

### Accuracies achieved

**Figure 4:**
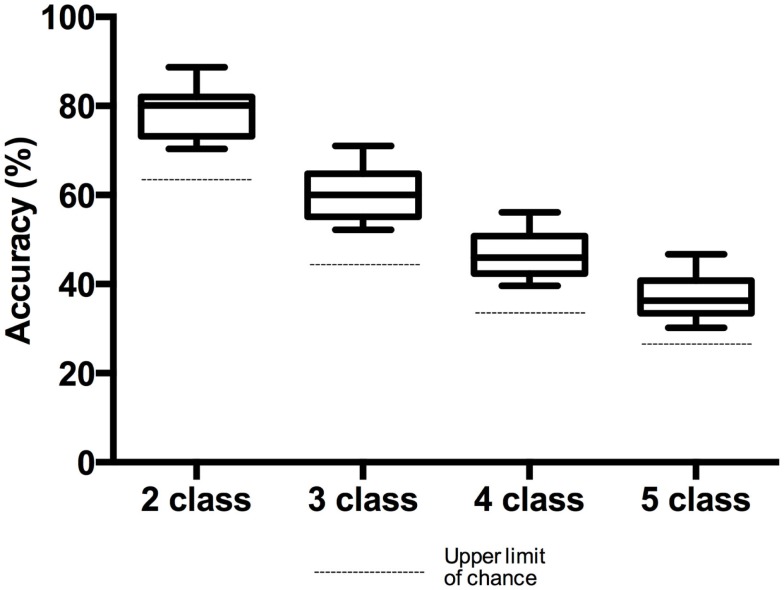
**Box plot of accuracies for the two-, three-, four-, and five-class problems**. Whiskers extend from min to max value. The dashed line below each box plot shows the upper limit of chance accuracy for each classification problem.

The accuracies achieved for the two-, three-, four-, and five-class problems are shown in Figure 4. Average classification accuracies of 78.4 ± 5.7, 60.5 ± 6.%, 46.7 ± 5.7, and 37.2 ± 5.4%, were achieved for the two-, three-, four-, and five-class problems, respectively.

All participants exceeded the 70% threshold for the two-class problem, and two participants (P3 and P5) exceeded the 70% threshold for the three-class problem.

All participants exceeded chance levels for all *n*-class problems. Theoretically, for 2, 3, 4, and 5 classes, the chance level accuracies are 50, 33, 25, and 20%, respectively. However, when the number of trials is less than infinity, the chance levels are those values plus or minus a confidence interval, given a value α (Mueller-Putz et al., 2008). Using the equation presented in (Mueller-Putz et al., 2008), the confidence limits for randomized class labels were calculated (Table 2). For the classifier accuracy to be statistically greater than chance, accuracies must exceed the upper confidence limit. In all cases, the classification of participant data with randomized class labels fell within the confidence limits of chance.

**Table 2:** **Chance levels and corresponding confidence limits for the two-, three-, four-, and five-class problems given 60 trials per class and α = 0.05**.

Class	Chance level (%)	Confidence limits (%)
2	50	(36.8, 63.2)
3	33.4	(23.2, 44.2)
4	25	(17.2, 33.6)
5	20	(14.3, 26.5)

*The Bonferroni correction was used to account for the multiple task-subsets explored (Kaltenbach, 2012). Therefore, adjusted α values of 0.0033, 0.0025, 0.0033, and 0.0083 were used for the two-, three-, four-, and five-class problems, respectively*.

### Task frequency analysis

**Figure 5:**
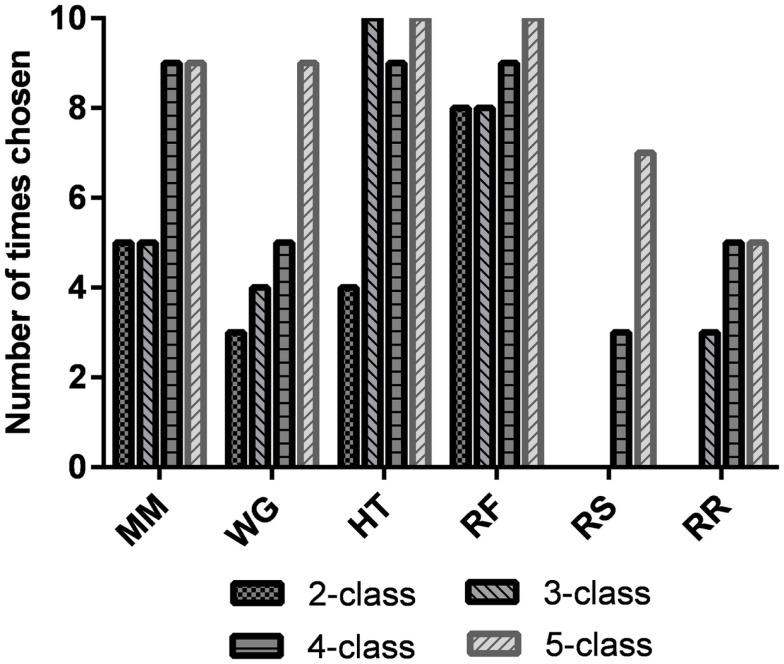
**Number of times that each task was chosen as the best task for a participant for the two-, three-, four-, and five-class problems**. It should be noted that since there are 10 participants, if a task is chosen 10 times, then it was chosen for all the participants. MM, mental math; WG, word generation; HT, happy thoughts; RF, relaxing with focus; RS, relaxing with slow counting; and RR, unconstrained rest.

The best task pairs for each of the participants and for all *n*-class problems are shown in Table 3. The most common combination of tasks chosen for the two-, three-, four-, and five-class problems are shown in the last row of Table 3. Additionally, the individual task frequencies for each of the *n*-class problems are shown in Figure 5. The most frequently chosen tasks over all classification problems were happy thoughts (HT) and relaxing with focus (RF).

**Table 3:** **Best task combinations for two-, three-, four-, and five-class problems**.

Participant	Two-class	Three-class	Four-class	Five-class
1	WG RF	WG HT RF	MM WG HT RF	MM WG HT RF RS
2	MM RF	WG HT RF	MM WG RF RS	MM WG HT RF RS
3	HT RF	WG HT RR	WG HT RS RR	MM WG HT RF RR
4	MM WG	MM WG HT	MM WG HT RF	MM WG HT RF RS
5	MM RF	MM HT RF	MM HT RF RR	WG HT RF RS RR
6	HT RF	HT RF RR	MM HT RF RR	MM WG HT RF RR
7	WG RF	MM HT RF	MM WG HT RF	MM WG HT RF RS
8	MM HT	MM HT RF	MM HT RF RR	MM WG HT RF RS
9	HT RF	HT RF RR	MM HT RF RR	MM WG HT RF RR
10	MM RF	MM HT RF	MM HT RF RS	MM HT RF RS RR
Most common	HT&RF and MM&RF	MM&HT&RF	MM&HT& RF&RR	MM&WG&HT& RF&RS

*MM, mental math; WG, word generation; HT, happy thoughts; RF, relaxing with focus; RS, relaxing with slow counting; and RR, unconstrained rest*.

### Correlations of accuracy with user characteristics

Table 4 shows the correlations between assessed criteria and the two-class classification accuracies over all participants. Strong positive correlations were found between accuracy and concentration (ρ = +0.634, *p* < 0.05), ease of session (ρ = +0.653, *p* < 0.05), and enjoyment (ρ = +0.550, *p* < 0.10), while accuracy and verbal IQ were strongly negatively correlated (ρ = −0.749, *p* < 0.05). It is noted that verbal IQ scores ranged from 80 to 116. Additionally, a moderate non-significant negative correlation was found between accuracy and tiredness before the session. Finally, weak to no correlation was found between accuracy and gender, as well as between accuracy and handedness. However, it is noted that there were six female participants and eight right-handed participants. As a result of a homogeneous age range (seven of the participants were in their twenties), no correlation analysis between age and accuracy was conducted.

**Table 4:** **Correlations between two-class accuracies and user characteristics (α = 0.1)**.

	Correlation with two-class accuracy (ρ)	*p*-value (*p*)
Verbal IQ	Pearson’s ρ = −0.749	**0.020**
Enjoyment	Pearson’s ρ = 0.550	**0.100**
Tiredness	Pearson’s ρ = −0.449	0.193
Concentration	Pearson’s ρ = 0.634	**0.049**
Ease of session	Pearson’s ρ = 0.653	**0.041**
Gender	Spearman’s ρ = −0.221	0.540
Handedness	Spearman’s ρ = 0.015	0.968

*Bold indicates p < 0.1*.

## Discussion

### Comparison of classification accuracies

The average two-class accuracy achieved in this work (78.4%) appears to be on par with the high-end of those reported in other NIRS–BCI studies over the PFC (Power et al., 2010, 2011; Schudlo et al., 2013). As expected, when moving beyond binary classification there was a significant drop in the accuracies achieved as a result of the increasing complexity of the classification problem. Overall, our results show promising progress toward distinguishing three and four mental tasks using an NIRS–BCI over the PFC. For most participants, the accuracies achieved in this study are still not sufficient for real-world BCI use; however, two participants (P3 and P5) were able to exceed the 70% threshold for a three-class problem. It is noted that these two participants had the two highest enjoyment, concentration, and reported ease-of-use ratings, and had two of the three lowest verbal IQ scores.

The average three- and four-class accuracies achieved in this work (61 and 47%) appear to be on par with the high-end of those reported in other multi-class NIRS–BCI studies over the PFC (Hirshfield et al., 2009; Power et al., 2012a; Herff et al., 2013a,b), namely average three-class accuracies of 50% (Herff et al., 2013a), 54% (Hirshfield et al., 2009), and 56% (Power et al., 2012a), and a four-class accuracy of 45% (Herff et al., 2013a). Moreover, contrary to our study, it appears that in NIRS–BCI literature, to date, no participant was able to exceed the 70% threshold for a three-class problem. Additionally, to the best of our knowledge, no other NIRS–BCI study has attempted to differentiate five mental tasks over the PFC.

Note that the accuracies achieved for three- and four-class NIRS–BCIs over the PFC are still much lower than those achieved for EEG-BCIs and NIRS–BCIs over the motor cortex (Palaniappan et al., 2002; Dobrea and Dobrea, 2009; Gupta et al., 2009; Chai et al., 2012; An et al., 2013; Shin and Jeong, 2014). This is in line with similar trends of lower accuracies in two-class NIRS–BCI studies over the PFC (Power et al., 2011), when compared to two-class EEG-BCI studies (Nai-Jen and Palaniappan, 2004) and NIRS–BCI studies over the motor cortex (Sitaram et al., 2007). However, as previous researchers have pointed out, there are numerous advantages to using NIRS over the PFC. Specifically, the headband is not intrusive and requires minimal set-up time when compared to both EEG-BCIs and NIRS–BCIs over the motor cortex (Bauernfeind et al., 2008; Power et al., 2012a; Herff et al., 2013b; Kopton and Kenning, 2014). Additionally, motor tasks may not be suitable for all users, such as our target population of clients with motor impairments (Curran et al., 2004). When conducting NIRS–BCI measurements over areas with hair, there are additional challenges, including the integrity of the optode–skin contact and attenuation of light by hair (Lloyd-Fox et al., 2010). To combat this, spring loaded sources and detectors can be used, but these have been shown to be uncomfortable (Lloyd-Fox et al., 2010), with several participants dropping out of studies due to headset discomfort (Suzuki et al., 2010; Cui et al., 2011). On the other hand, it appears that users found the NIRS headband in this study to be comfortable. Participants evaluated the post-session statement “The headgear was comfortable” at an average rating of 5.3 ± 0.9 on the 7-point Likert-type scale, indicating that on average participants agreed with this statement. Moreover, none of the participants reported the headset to be uncomfortable (no rating <4).

### Correlation between user characteristics and accuracy

Although correlation analyses were conducted on only 10 participants, several interesting trends warrant further exploration.

#### Increasing Accuracy with Decreasing Verbal IQ

Contrary to the EEG-BCI results by Hammer et al. (2012), who found no correlation between accuracy and non-verbal intelligence, in this study we found a strong negative correlation between accuracy and verbal intelligence. This correlation may seem surprising at first; however, upon further analysis, it appears that this trend may be attributable to task difficulty and neural efficiency.

In addition to the correlation between IQ and accuracy, a negative correlation was found between verbal IQ and the range (max–min) of HbO concentrations of the chosen tasks (ρ = −0.603, *p* < 0.1). This indicates that in general, individuals with lower IQ elicited larger overall changes in their hemodynamic activity. A possible explanation for this is that individuals with lower verbal IQ scores tend to elicit stronger, more consistent changes in neuronal hemodynamic activity when performing mental tasks since they find them to be more challenging.

In literature, the relationship between intelligence and hemodynamic brain activity is still widely debated, and is often referred to as the “neural efficiency debate” (Graham et al., 2010). Similar to findings in this study, several researchers reported that a decrease in IQ or skill was associated with an increase in brain activation. An MRI study conducted by Graham et al. found that participants with average IQ showed greater PFC activation during response selection than did high IQ participants. The authors argued that the participants with high IQs invoked more resource-efficient cognitive strategies resulting in less activation (Graham et al., 2010). Additionally, a positron emission tomography (PET) study by Haier et al. (1992) concluded that there was an inverse correlation between neural activity and verbal IQ scores in several brain regions, including areas of the frontal cortex. Moreover, an MRI study by Rypma et al. showed that during a working memory task, higher performing participants had overall less PFC activation than lower performing participants. This study also found that higher performing participants exhibited a larger increase in activation with increased task difficulty (Rypma and D’esposito, 1999; Rypma et al., 2002). Collectively, these results reveal a relationship between task difficulty and IQ, and motivate further exploration of personalized task difficulty levels for each participant based on IQ scores.

#### Increasing Accuracy with State of Mind Changes

We found a strong significant positive correlation between accuracy and each of concentration and reported ease. These results appear to be in line with EEG-BCI literature. Specifically, Hammer et al. (2012) noted a positive correlation between accuracy and attention (ρ = 0.5, *p* = 0.02), while Nijboer et al. (2008) documented a positive correlation between accuracy and mastery confidence (*b* = 0.578, *p* < 0.05).

Additionally, we found a strong significant positive correlation between accuracy and task enjoyment. This finding also resonates with EEG-BCI literature. Friedrich et al. (2013) uncovered a positive correlation between accuracy and self-reported task enjoyment (ρ = 0.3, *p* < 0.1), while Nijboer et al. (2008) cited a positive correlation between accuracy and mood (*b* = 0.498, *p* < 0.05).

Finally, we found a moderate, but not significant, negative correlation of accuracy with tiredness. This trend appears to be in contrast to the previous EEG-BCI finding of increased accuracy with decreased sleep in a P300 BCI (*p* < 0.05) (Guger et al., 2009). However, due to the very different neural mechanism involved in using a P300 BCI, this inconsistency is not surprising.

Overall, these findings motivate future research to enhance NIRS–BCI accuracy via training for confidence and concentration, and maximizing enjoyment while minimizing fatigue.

## Limitations and Future Work

This study was conducted under controlled conditions which included a dimly lit room free of distractions. Future studies should be conducted in more practical environments in order to assess the functionality of the BCI in less than optimal conditions.

Second, the study was conducted on able-bodied participants. For use as an access technology for individuals with severe motor impairments, the results obtained likely do not reflect the performance of this target population. Further research and testing on a clinical population is necessary before conclusions about the effectiveness of multi-class BCIs can be made.

Third, when using NIRS as an access modality for a BCI, there is the potential for systemic contributions to the signal (Takahashi et al., 2011). Although systemic noise is likely present, it has been shown that the majority of the signal originates from the cerebral cortex (Kirkpatrick et al., 1995; Hoshi et al., 2011; Funane et al., 2014). Moreover, the cortical component has been shown to be non-trivial; strong correlations have been reported between NIRS and fMRI signals (Cui et al., 2011; Sasai et al., 2012; Sato et al., 2013) and between NIRS and EEG signals (Moosmann et al., 2003; Koch et al., 2008; Roche-Labarbe et al., 2010; Talukdar et al., 2015).

Fourth, in addition to the user characteristics described in this work, several other factors may be correlated to NIRS–BCI accuracy. Specifically, future work should explore the correlation of anatomical features with accuracy, such as scalp-cortex distance and frontal sinus volume, as these have been shown to be correlated with NIRS signal quality (Haeussinger et al., 2011). Other future directions include correlation analysis with respect to task performance, as well as the exploration of within-subject correlations on a per-session basis.

Finally, this study was conducted offline and over a relatively short period of time, with only five data collection sessions. It is possible that when moving online (with the inclusion of real-time performance feedback) and when conducting studies over longer periods of time (with the possibility of learning and habituation), performance and the correlation of performance with user characteristics may change. Further research on online and long-term trends is necessary.

## Conclusion

This study explored the use of personalized mental tasks to increase the number of outputs of an NIRS–BCI. Average classification accuracies of 78.4 ± 5.7, 60.5 ± 6.6, 46.7 ± 5.7, and 37.2 ± 5.4% were attained for the two-, three-, four-, and five-class problems, respectively. All participants exceeded the 70% threshold for the two-class problem, and most notably, two participants were able to exceed an accuracy of 70% for the three-class problem.

Accuracy positively correlated with ease of session, ease of concentration, and enjoyment, and negatively correlated with verbal IQ. Future multi-class NIRS–BCI research ought to consider the development of training paradigms for maximizing user concentration, enjoyment, and confidence, as well as personalization of task difficulty based on IQ.

Overall, this research provides an incentive for further exploration of multi-class NIRS–BCIs, as well as continued research on the user characteristics that affect classification accuracies.

## Conflict of Interest Statement

The authors declare that the research was conducted in the absence of any commercial or financial relationships that could be construed as a potential conflict of interest.
